# Short Comings

**Published:** 1857-02

**Authors:** 


*Short Comings.*—The present number is a scape-goat. It goes out into the wilderness heavy with the sins and omissions of the past three months —months which have been pretty fully occupied. Delayed communications have finally found entrance by the simple process of excluding selected matter, and reviews by dint of contraction into mere book-notices have also been squeezed in between our covers. To book-publishers and correspondents we promise better usage in future.

In our next number we shall give reviews of Dunglison's Human Physiology, Taylor's Medical Jurisprudence, Churchill on Children, Barton's Cause and Prevention of Yellow Fever, Laycock on Medical Observation, Rigby on Female Diseases, Bartlett on Fevers, and Holland's Medical Notes.

Editorially we are also behind hand. Two or three of our cotemporaries are attacking us on all sorts of grounds, and we are compelled to sit meekly and endure it. Wrath does not—with us—keep well when bottled up for any length of time, and we find ourselves losing all power of effervescence. We hope for better things.

